# The complexity of clinically-normal sinus-rhythm ECGs is decreased in equine athletes with a diagnosis of paroxysmal atrial fibrillation

**DOI:** 10.1038/s41598-020-63343-7

**Published:** 2020-04-22

**Authors:** Vadim Alexeenko, James A. Fraser, Mark Bowen, Christopher L.-H. Huang, Celia M. Marr, Kamalan Jeevaratnam

**Affiliations:** 1 Faculty of Health and Medical Sciences, University of Surrey, Guildford, GU2 7AL United Kingdom; 2 Physiological Laboratory, University of Cambridge, Cambridge, CB2 3DY United Kingdom; 3 Faculty of Medicine & Health Sciences, University of Nottingham, Nottingham, NG7 2UH United Kingdom; 4 Rossdales Equine Hospital and Diagnostic Centre, Exning, CB8 7NN Suffolk, United Kingdom; 5 Division of Cardiovascular Biology, Department of Biochemistry, University of Cambridge, Cambridge, CB2 1QW United Kingdom

**Keywords:** Arrhythmias, Information technology

## Abstract

Equine athletes have a pattern of exercise which is analogous to human athletes and the cardiovascular risks in both species are similar. Both species have a propensity for atrial fibrillation (AF), which is challenging to detect by ECG analysis when in paroxysmal form. We hypothesised that the proarrhythmic background present between fibrillation episodes in paroxysmal AF (PAF) might be detectable by complexity analysis of apparently normal sinus-rhythm ECGs. In this retrospective study ECG recordings were obtained during routine clinical work from 82 healthy horses and from 10 horses with a diagnosis of PAF. Artefact-free 60-second strips of normal sinus-rhythm ECGs were converted to binary strings using threshold crossing, beat detection and a novel feature detection parsing algorithm. Complexity of the resulting binary strings was calculated using Lempel-Ziv (‘76 & ‘78) and Titchener complexity estimators. Dependence of Lempel-Ziv ‘76 and Titchener T-complexity on the heart rate in ECG strips obtained at low heart rates (25–60 bpm) and processed by the feature detection method was found to be significantly different in control animals and those diagnosed with PAF. This allows identification of horses with PAF from sinus-rhythm ECGs with high accuracy.

## Introduction

Atrial fibrillation (AF) is a common arrhythmia in horses and it frequently occurs in the absence of gross structural abnormalities of the heart. The prevalence of this condition is estimated to be up to 2.3%^1,2^ although it might be higher in breeds predisposed to it^3,4^. Not only does this condition adversely affect the race performance of equine athletes^2,5^, but it may also promote more grave consequences such as arrhythmia-induced cardiomyopathy^6^, thereby reducing ventricular function by promoting heart failure, or leading to death by ventricular fibrillation^7,8^. Although appropriate ECG-based screening programmes aimed to prevent sudden cardiac deaths have been developed for human athletes^9^, such screening of equine athletes is extremely infrequent. This may be in part due to the absence of an agreed interpretation criteria for the equine ECG and the somewhat subjective nature of its interpretation. As a result, the inter-evaluator agreement in equine ECG analysis can be poor, especially for strips recorded at high heart rates and processed by inexperienced assessors^10^.

The use of ECG for diagnostic purposes might be changed by the introduction of objective measures and automatization of its analysis. The recent advent of machine learning techniques for ECG interpretation might be expected to contribute to this. The power of such techniques was demonstrated in a very recent study by Attia *et al*.^11^, which used a convolutional neural network to analyse the recordings from leads I, II and V1-6. Their algorithm was able to detect the presence of AF from a single normal sinus rhythm ECG with sensitivity and specificity close to 0.8. Although this study does not directly provide insights into the physiological mechanisms that are detected by the “black box” machine learning algorithms, it is a powerful indicator that there are physiological changes in apparently normal sinus rhythm ECGs which might be used for early arrhythmia detection.

It might be expected that non-linear analysis methods could provide a feasible and more mechanistically transparent approach to detect these occult ECG changes^12^. We have recently demonstrated that disorders associated with anomalous generation and propagation of electrical signals in the equine heart could be assessed using telemetric ECGs and signal complexity estimation techniques^13^. These techniques were previously shown to be sensitive to the irregularity of various bioelectrical signals^14–17^, including ECG^18–21^. The inherently chaotic nature of cardiac electrical activity^22^ makes complexity analysis an appropriate tool to assess its stochasticity and detect alterations that might be associated with arrhythmias.

The origin of complexity estimation techniques can be traced to the seminal work of Kolmogorov^23^. He suggested that the disorderliness (complexity) of a string of symbols could be described as the length of the shortest possible computer program capable of generating such a string. As Kolmogorov’s complexity is an incomputable metric by definition, a number of different techniques to estimate it have been developed. Arguably, the most influential contribution was that of Lempel and Ziv who demonstrated that complexity can be feasibly linked to the gradual build-up of new patterns along a given sequence of symbols^24^. The key idea of Lempel and Ziv’s initial method (usually abbreviated as LZ76) is to decompose the source string of symbols to a vocabulary of unique substrings (“factors”) which are sufficient to rebuild the source string by a machine performing copy and insertion operations^25^. The vocabulary size is then directly proportional to the complexity of the source string. Detailed explanation and discussion of LZ76 decomposition may be found in previously published works^24–27^.

A slightly modified, faster version of Lempel-Ziv decomposition, which was developed in 1978 for data compression (LZ78), could also be used for complexity estimation^28^. A common feature of both Lempel-Ziv parsers is incomplete processing of the final part of the analysed string. This might lead to additional variability in the analysis of short strings^29,30^. Although such error could be decreased by analysing longer strings, this is not always possible, and nor is it desirable as it limits the temporal resolution of the analysis method. Such error might also be limited by using algorithms which are capable of more complete parsing of the source data, as proposed by Titchener, for example^31^. In our previous work^13^ we demonstrated that the behaviour of a Titchener complexity estimator in ECG analysis produced result very similar to LZ76, while the LZ78 estimator produced different results from either LZ76 and Titchener ones. We also estimated that analysis of 60-sec ECG strips might be a reasonable compromise between the competing requirements of having a sufficient amount of data with a low coefficient of variation, whilst keeping the strip length short enough to keep the observed parameters stationary during data collection. As the recordings used for our previous study on the feasibility of complexity analysis of telemetric equine ECGs were collected from healthy subjects only, we re-used these data files as a control cohort alongside ECGs from horses with a diagnosis of AF that were collected over the same time-frame and under the same conditions but had not previously been analysed.

Both Lempel-Ziv and Titchener complexity estimators require symbolic strings to process, therefore necessitating data preconditioning and coarse-graining to convert floating-point ECG data into a string of symbols. The preconditioning steps typically include low-pass or band-pass filtering for baseline wander correction and resampling to a pre-defined sampling frequency to a standardized rate. There is no consensus on the parameters of such filters, the desirable sampling rate, or the subsequent coarse-graining method. In previous work, two choices of coarse-graining have been employed: (1) threshold-crossing (TC), in which values exceeding a threshold are converted to a value of one, while values below the threshold are set to zero, and (2) beat detection (BD), in which the value at the R peak is assigned as one and the remaining values are set to zero. The present work also introduces and assesses a third coarse-graining scheme in which several specific ECG features of the heartbeat are detected (feature detection, FD). These approaches might be feasibly regarded as quantifying primarily the variability of electrical activity close to the isoelectric line (TC), variability in the electrical activity of the pace-making nodal pathway (BD) and the overall variability of relative ECG feature durations and timings (FD).

Thus, the present work assesses the ability of various forms of complexity analysis to detect paroxysmal atrial fibrillation in horses, in order to investigate the most promising methods to screen for this arrhythmia.

## Results

### ECG complexity is altered by PAF

Our previous study^13^ has demonstrated that there is a relationship between ECG complexity and heart rate. While there is a strong positive linear correlation between heart rate and complexity at heart rates in 25–60 bpm range, such dependence is less pronounced at higher heart rates where complexity values become very variable. To limit the errors caused by complexity variability at higher heart rates we limited analysis to strips in a 25–60 bpm range. Since a number of control subjects did not provide any 60-second artefact-free normal sinus rhythm ECGs in this range, some of them were excluded giving a final data set of 51 subjects in the control group, 10 in the PAF group. There was no significant difference in heart rates between the control group (39.3 ± 7.7 bpm) and PAF group (37.8 ± 5.6) bpm.

**Figure 1:**
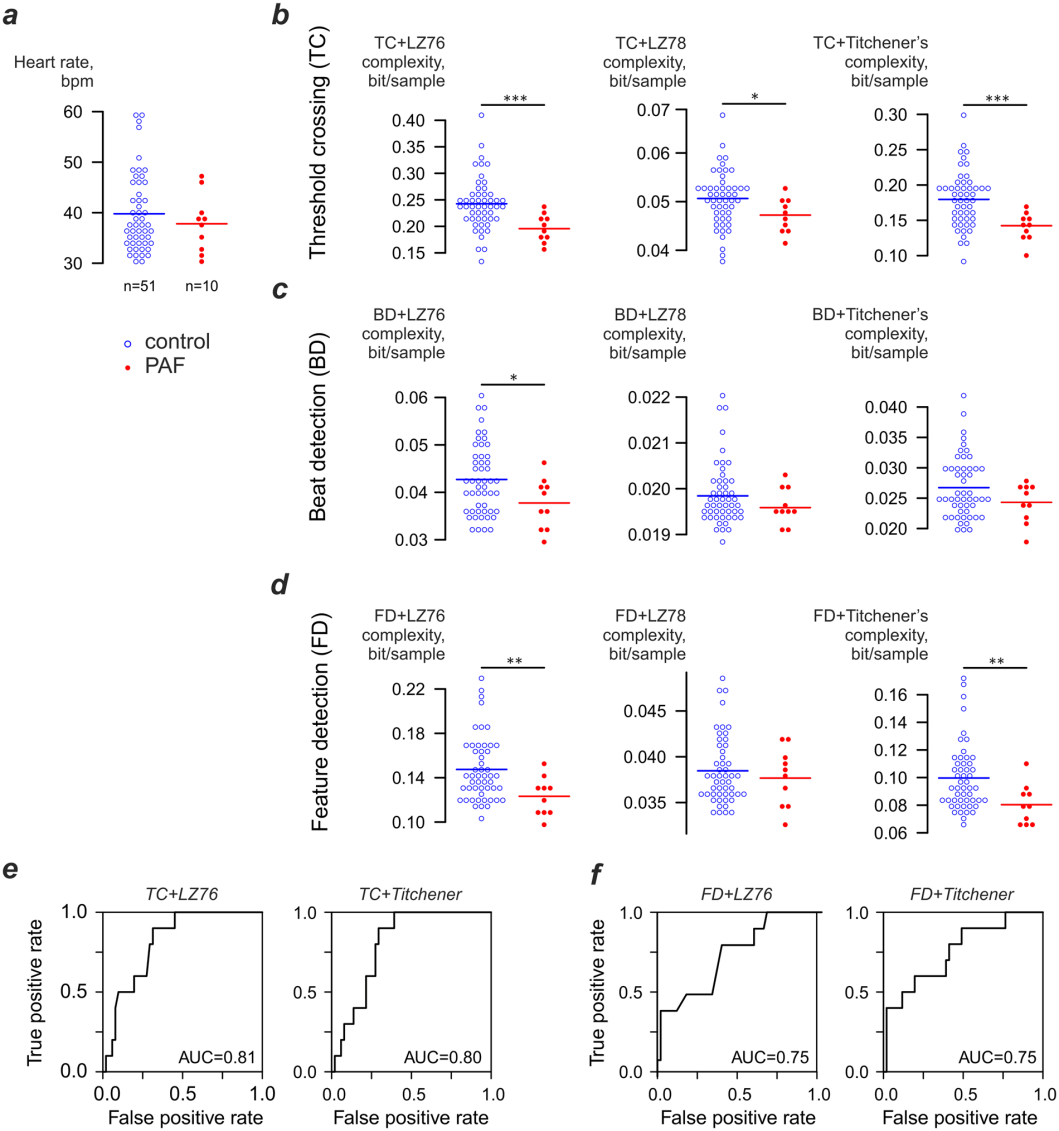
Heart rate and complexity of ECG strips obtained using different coarse-graining and complexity estimation techniques. (**a**) Heart rate of the analysed ECG strips. (**b**) Complexity of ECG strips processed using threshold-crossing coarse-graining technique. Mean values for the groups indicated by horizontal bars in all panels. (**c**,**d**) Complexity of ECG strips processed using beat- detection and feature detection coarse-graining techniques. (**e**) Receiver operating curve analysis for the LZ76 and Titchener complexity-based detection of PAF using the threshold crossing coarse-graining. (**f**) Receiver operating curve analysis for the LZ76 and Titchener complexity-based detection of PAF using the feature detection coarse-graining.

To evaluate the link between ECG complexity and PAF we used the first 60-sec strip obtained from each horse that was of a quality suitable for the analysis. All strips selected for analysis were processed using three complexity estimators (LZ76, LZ78, Titchener) and three coarse- graining methods (TC, BD, FD, see Fig. 1). Threshold crossing coarse-graining in conjunction with LZ76 demonstrated significant (p = 0.003) decrease of ECG complexity in the PAF group (0.196 ± 0.027 bit/sample) compared to controls (0.243 ± 0.050 bit/sample). Similar results were demonstrated by the combination of TC coarse graining and Titchener complexity: ECG complexity in the PAF cohort dropped to 0.142 ± 0.039 from 0.180 ± 0.039 bit/sample (p = 0.001). Less significant difference was observed for LZ78 analysis of threshold-crossing data: PAF group complexity decreased to 0.047 ± 0.0033 from 0.050 ± 0.0057 bit/sample in controls (p=0.02). The performance of PAF predictions based on these estimators was assessed using receiver operating curve analysis, which produced area under curve (AUC) values close to 0.8 (Fig. 1e) for both LZ76 and Titchener complexity estimators.

Using the same three complexity estimators with BD coarse-graining method produced the significant difference only for LZ76 complexity estimator: in controls average complexity was 0.043 ± 0.0074 bit/sample and in cases 0.0378 ± 0.0054 (p=0.02). Both LZ78 and Titchener complexity failed to demonstrate any significant differences between cases and controls (Fig. 1c). Somewhat more promising results were obtained with the FD coarse-graining technique (Fig. 1d), where LZ76 and Titchener complexity estimators revealed significantly lower complexity values for the PAF group than for the controls (LZ76: controls 0.147 ± 0.029 bits/sample, cases 0.123 ± 0.018, p = 0.003; Titchener: controls 0.0997 ± 0.024 bits/sample, cases 0.080 ± 0.014 bits/sample, p = 0.003). Receiver operating curve analysis produced results similar to threshold-crossing coarse-graining. The AUC values were 0.75 for both complexity estimators.

### Heart rate influences ECG complexity

To assess whether the relationship between complexity and heart rate might be influenced by PAF we evaluated the relationship between heart rate and ECG complexity in both control and PAF cohorts and for each combination of coarse-graining technique and complexity estimator. It was found that TC coarse-graining exhibited low dependence on the heart rate for all complexity estimators (Spearman r of 0.41–0.44); and we decided to exclude this coarse graining technique from further consideration. As LZ78 estimator in conjunction with both BD and FD coarse graining produced similar complexity values for both controls and cases and thus was excluded it from further consideration as well.

In the remaining combinations of BD and FD coarse graining and LZ76 and Titchener complexity estimators, dependence of complexity on the heart rate was very pronounced. Spearman r_s_ was 0.77 for the BD + LZ76 and 0.82 for the BD + Titchener combination. Even greater dependence was found in FD processed data, with r_s_ being 0.82 for the FD + LZ76 pair and r_s_ = 0.97 for the FD + Titchener combination. The most important observation was that in the combination of BD and FD coarse-graining with LZ76 and Titchener complexity estimators, the dependence of complexity and heart rate was not only linear but also influenced by PAF.

**Figure 2:**
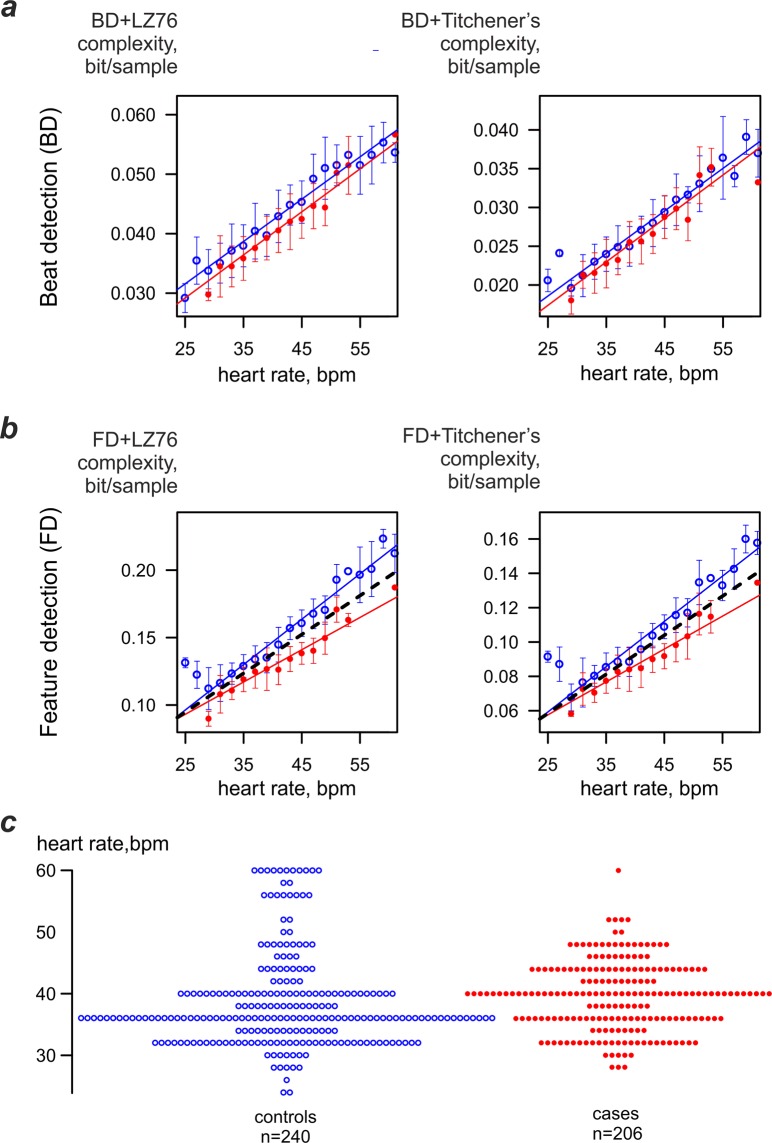
Dependence of ECG complexity obtained by different estimators and different coarse-graining techniques on the heart rate. (**a**) Beat-detection coarse-graining. (**b**) Feature-detection coarse graining. In both graphs thin lines show the linear fit for the entire data set provided by a cohort (controls n = 240, cases n = 206); dashed line shows the suggested threshold to differentiate PAF cases and controls. Threshold-crossing data not shown due to the weak correlation between the heart rate and complexity(r < 0.5). (**c**) Heart rates in the analysed ECG strips.

To assess that relationship, ECG strips for both subject cohorts were grouped by a single bpm and the dependence of mean complexity on the heart rate was plotted for each group. Figure 2 shows the dependence of complexity values for both cases and controls. It includes data collected in all subjects over long periods of time. While there was little influence of PAF on the BD-processed ECGs (Fig. 2a), there was very noticeable effect of PAF on the FD-processed strips (Fig. 2b). The relationship between heart rate and complexity was steeper in the control group both for LZ76 (3.4 · 10^−3^ vs 2.4 · 10^−3^ bit*bpm/sample; standard errors 1.0 · 10^−4^ and 1.4 · 10^−4^ bit*bpm/sample correspondingly) and for Titchener complexity estimators (2.6 · 10^−3^ vs 1.9 · 10^−3^ bit*bpm/sample; standard errors 8.7 · 10^−5^ and 1.2 · 10^−4^ bit*bpm/sample correspondingly). To quantify this observation, analysis of covariance was used to compare the relationship of complexity to heart rate for each cohort. There was a significant interaction (p < 0.001) in the relationship of both LZ76 and Titchener complexities to cohort and heart rate. For both LZ76 and Titchener complexity estimators, the slope between heart rate and complexity was significantly different for control and PAF groups (p < 0.001).

### Combined complexity/heart rate metric might reveal the presence of pro-arrhythmic background

**Figure 3:**
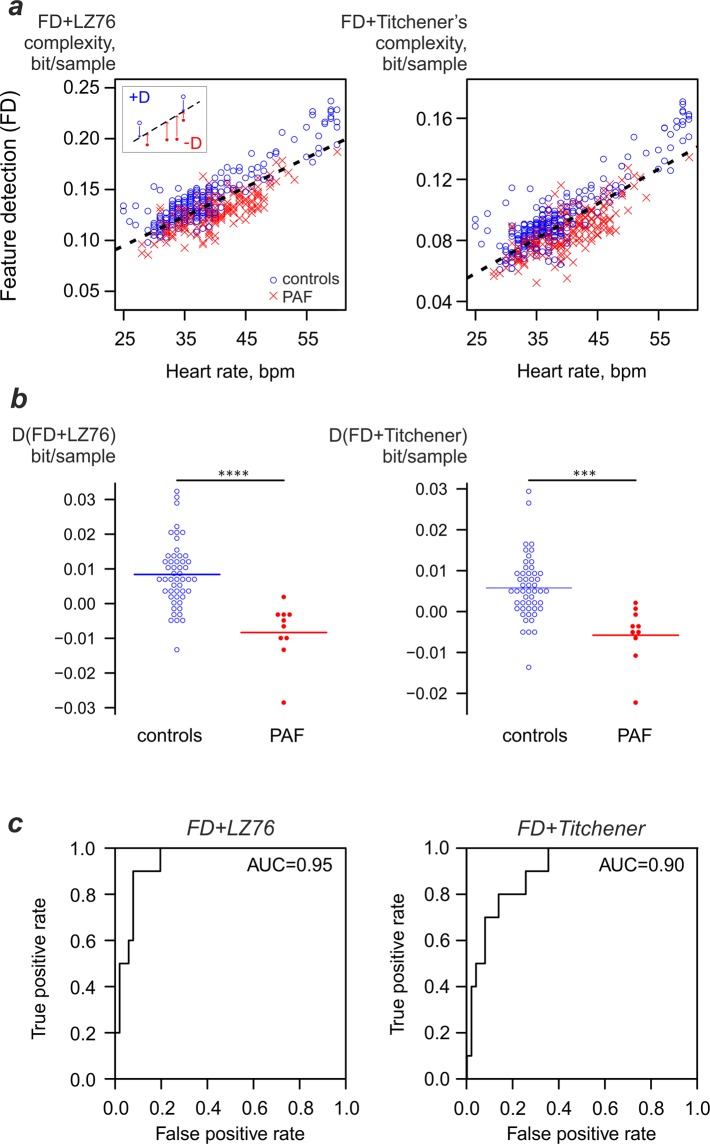
Heart-rate adjusted complexity measure based on feature detection coarse-graining may detect the pro-arrhythmic background. (**a**) Dependence of complexity values on heart rate. Threshold used for detection is shown as dashed black line. The average vertical distance from the subject’s data points to a threshold line is used as a discriminator (inset). (**b**) Per-patient combined complexity/heart rate metric values. (**c**) Performance of PAF detection using the metric.

The dependency of LZ76 and Titchener complexities on heart rate for FD coarse-grained data suggested that accounting for heart rate could increase the sensitivity of PAF detection using complexity analysis. To obtain the prediction, average vertical (complexity) distances from the threshold line to the individual data points (D(FD + LZ76) and D(FD + Titchener)) were calculated for each horse (Fig. 3a). Positive values corresponded to complexity values above the threshold line and negative values indicated complexity being below it (see inset). The median number of analysed strips was five per horse. The average D(FD + LZ76) for the control group was 0.0084 ± 0.0092 bit/sample (n = 51) and for the PAF group it was −0.00834 ± 0.00849 (n = 10, p = 7*10^-5^). At the same time, average D(FD + Titchener) for the control group was 0.00578 ± 0.00751 bit/sample, while for the PAF group it was −0.00578 ± 0.00711 (p = 0.00043); see Fig. 3b. The corresponding receiver operating curves for differentiation between PAF and controls (Fig. 3c) show that such a combined discriminator has superior performance compared to the method relying on complexity only (compare Figs. 1e,f and 3c).

To verify the dependence of the performance of this metric on the number of strips, we performed several analyses artificially limiting the maximum number of strips included in the analysis. Such artificial limitation did not have noticeable influence on the performance of the metric. In the range of four to ten strips the area under curve remained close to 0.95 for LZ76 and 0.90 for Titchener complexity estimator. Even if the analysis was limited to a single strip, the area under curve was close to 0.75 for both complexity estimators.

## Discussion

This study introduces a novel approach to the detection of paroxysmal atrial fibrillation in horses, suggesting that it may be detected from sinus rhythm ECGs. We suggest that feature detection ECG parsing, combined with heart-rate corrected complexity estimation (either LZ76 or Titchener) might be considered as a promising tool for PAF diagnosis (Fig. 3c). This tool produced a highly accurate automated discriminator between PAF and controls using sinus-rhythm ECGs (AUC exceeding 0.9 for both complexity estimators). A key advantage of the proposed method is that, unlike manual ECG analysis or other automated methods of AF detection based on estimation of signal stochasticity^32^, it does not require the actual fibrillation episode to occur during the ECG recording. However, this also means that such a technique does not actually document the episode of AF as an ultimate proof required for the diagnosis to be confirmed. Therefore, prolonged ECG monitoring would still be required to obtain such an unambiguous proof^33^.

Our approach is similar to the recently published work by Attia *et al*.^11^, in that it assesses PAF risk from sinus rhythm ECGs. The present approach shows similar or greater accuracy to the machine-learning approach in that study, albeit in a much smaller cohort. However, the use of a markedly different approach to ECG analysis provides the interesting possibility that ECG complexity measures could be added as determinants to train machine learning algorithms such as that described by Attia *et al*., potentially combining the predictive powers of each technique.

Our study demonstrates that coarse-graining method and complexity estimator choice has an important influence on the performance of ECG complexity analysis. Thus, although LZ76 and Titchener complexity estimators show significant complexity difference between PAF cases and controls in conjunction with TC and FD coarse-graining techniques, the observed effect size was not sufficient to consider these approaches alone as potential diagnostic methods.

An interesting aspect of this study is that the none of the coarse-graining methods make use of any features defined by electrical activity of the atria other than pacemaking. This raises a question of whether the changes we observe are the manifestations of global alterations in rhythm generation and conductive properties of the equine heart due either to electrical remodelling or to alterations in autonomic balance^34^. The concept of “lone atrial fibrillation” has largely fallen out of use in human cardiology^35^, and, although the term can still be found in the literature, it is losing popularity in veterinary medicine. Our observations might be considered as additional evidence in favour of getting rid of it completely.

Our study has some limitations. The retrospective nature of this work limited availability of data. As a result, a proportion of control subjects had to be excluded from the study due to fast heart rates. The relatively small case cohort prevented any analysis of which features were providing the most useful information for PAF detection. We expect that a larger prospective study will be needed to elucidate this. It could be expected that the P wave onset might provide an unambiguously detected feature which might convey additional sensitivity for PAF detection. Correct identification of P waves is typically easy in high-quality resting ECGs without electrical noise from skeletal muscular activity, although the analysis of real-world clinical data might be problematic. It might also be expected that some other physiological parameters or biomarkers could be combined with complexity analysis to provide greater power; one might expect that age, weight and height of an animal could be considered for this purpose.

It might be expected that complexity-based PAF prediction method might even be capable of detecting a pro-arrhythmic background before the first actual arrhythmia episode, allowing for early detection of high-risk equine athletes. We hope that careful selection of the ECG features for complexity analysis might elucidate some specific properties of the ECG associated with other pathological alterations. We hope that a future prospective longitudinal study might elucidate the predictive properties of such analysis.

This study thus emerges with an analysis technique that requires artefact–free normal sinus rhythm ECG recorded at low to very moderately increased heart rates of 25–60 bpm. These simple requirements suggest that a fully automated method could be developed to detect PAF. Such a system might incorporate machine learning^36,37^ to select ECG recordings of sufficient quality, with subsequent non-linear analysis providing the diagnostic outcome.

We might also foresee that a similar study has to be conducted in humans to verify if an analogous approach might be valid cross-species. Unlike in horses, PAF in humans is known to be a major cause of ischemic stroke, and a rapid and sensitive method for PAF detection or prediction is considered to be one of the major problems in cardiovascular medicine.

## Materials and Methods

### Subject recruitment

Based on the ethical assessment review checklist by the Non-ASPA Sub-Committee at the University of Surrey, the study did not require an ethical review and received appropriate faculty level approval. Non-invasive ECG recordings were collected as part routine clinical work at Rossdales Equine Hospital and Diagnostic Centre (Newmarket, Suffolk, United Kingdom). All subjects were thoroughbred horses of racing age undergoing race training. For the control group were used recordings from 82 healthy horses not displaying clinically significant cardiac abnormalities on prior routine cardiovascular examination. This control cohort was previously used to evaluate the feasibility of complexity analysis of equine ECG^13^. The PAF group consisted of newly recruited 10 horses for which a diagnosis of PAF had been made previously by ECG recording. Only horses which had an ECG confirmation of PAF diagnosis were included in the study as cases. Sinus rhythm ECGs were obtained after these horses had spontaneously converted to normal rhythm.

### Data recording

Horses were atraumatically fitted with a telemetric ECG recorder (Televet 100, Engel Engineering Services GmbH, Germany). ECGs were recorded in continuous episodes lasting up to 22 hours. ECGs were primarily recorded at rest, but as is typical of equine ambulatory ECGs, these included a range of heart rates as horses respond to their environment. In 3 of 10 PAF cases ECGs, the recording included additional periods of exercise during relatively steady incremental heart rate. This equivalent alternative to an incremental pacing protocol has previously been applied in studies of cardiac function *in vitro*^38,39^. The Televet 100 recorder has signal bandwidth of 0.05–100 Hz and sampling rate of 500 Hz.

### Data preparation

**Figure 4:**
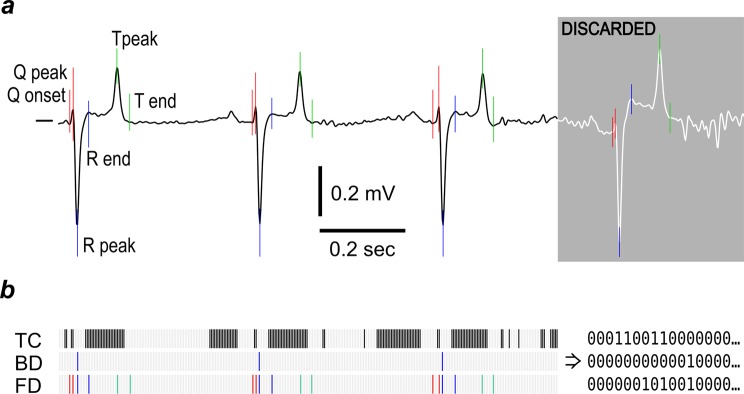
Methods of equine ECG coarse-graining. (**a**) Cardinal points selected for complexity analysis. The greyed part of the plot shows a typical part of the ECG that was rejected for low signal quality. (**b**) Coarse-graining methods used for complexity analysis: TC - threshold crossing; BD - beat detection; FD - feature detection. Zero values are shown as white bars, unitary values as black or coloured bars. Note that the FD method captures significantly more information on the waveform shape than BD while being less sensitive to noise than the TC method.

The original data files were exported to text-only format (comma-separated values, CSV) files using TeleVet software. The exported files were plotted using a custom R^40^ script and locations of artefact-free 60-sec segments were recorded by a human evaluator. Another R script was used to extract such segments, filter them using a zero-phase low-pass digital fourth order Butterworth filter and resample them to 125 Hz sampling frequency. A cut-off frequency of 40 Hz^41^, as widely used in medical practice, was chosen to eliminate the high-frequency noise. The resulting files (707 strips for control horses and 227 for PAF cases) were processed by a custom ECG parsing algorithm written in C++, which detected the onset and peak of the Q wave, and the peak and termination of the R and T waves in each heartbeat waveform (Fig. 4a). Briefly, this algorithm relies on the analysis of the first derivative of voltage to discover the approximate location of the R peak. Then, after precise location of this peak is established by a peak finding routine, the location of T and Q peaks is determined in a similar way using the approximate location of these features derived from the already-known R-R interval duration. Then the onset of the Q wave, the end of the S wave and the end of the T wave were determined as the points where the absolute value of the voltage derivative becomes less than a threshold value. Only the strips in the heart rate range of 25–60 bpm were considered for the further analysis (controls n = 241, cases n = 206).

For further analysis, the ECG signal was converted to binary strings using three methods, as shown in Fig. 4b. Threshold crossing (TC) set samples equal to or greater than the median value to one, with all remaining time points set to zero; beat detection (BD), set the value to one at the time of the R peak and all remaining values to zero; and feature detection (FD), set the value to one at the onset and peak of the Q wave and at the peak and termination of the R and T waves, with all remaining values set to zero.

### Complexity analysis

**Figure 5:**
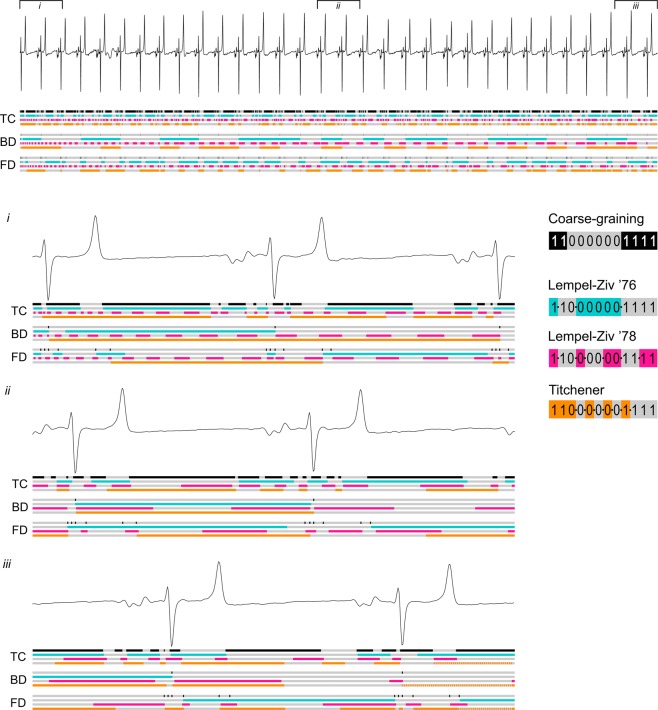
Behaviour of different complexity estimators in the analysis of equine ECG. In all panels: grey/black bar, the result of ECG granulation to a binary string, where black elements show the unitary values and grey elements show zeros. Other bars show the result of parsing of the binary string (cyan/grey- LZ76; pink/grey - LZ78; orange/grey – Titchener’s complexity). Borders between individual factors detected by the parser are indicated by alternating colours of each bar. Note the absence of unambiguous correlation between waveforms and factors.

Estimation of the binary string complexities was facilitated by a custom implementation of a complexity evaluator developed in C++ for the Linux operating system. The program simultaneously performs complexity analysis using three previously published methods: Lempel-Ziv ’76^24^, Lempel-Ziv ’78^28^ and Titchener T-complexity^31^. All three methods estimate the complexity of a symbolic string by identifying the number of sub-strings (factors) needed to build it by a computer capable of a certain limited set of operations. These methods differ by the algorithms of decomposition of the source strings to sub-strings and therefore produce different estimates for the same source data (Fig. 5). The detailed description of these methods may be found in the corresponding publications. To eliminate the dependency of complexity values on the length of the source string (n), Lempel-Ziv ’76 (abbreviated as LZ76) complexity values were normalised to the n/log_2_(n) value^25^. Lempel-Ziv ’78 (abbreviated as LZ78) values were normalised to sequence length. For T-complexity, average entropy values were used.

### Statistical analyses

Parametric data are expressed as mean ± standard deviation of mean. Statistical analyses and plotting were done using GNU R^40^. An unpaired two-sided t-test (using Welch’s correction for unequal variances) was used for two-group comparisons and ANOVA test with Tukey correction for multiple comparisons of groups, with significance between data sets accepted at p < 0.05.
